# DhMYB2 and DhbHLH1 regulates anthocyanin accumulation *via* activation of late biosynthesis genes in *Phalaenopsis*-type *Dendrobium*

**DOI:** 10.3389/fpls.2022.1046134

**Published:** 2022-11-15

**Authors:** Yachen Wang, Hantai Yin, Zongxing Long, Wenjuan Zhu, Junmei Yin, Xiqiang Song, Chonghui Li

**Affiliations:** ^1^ Key Laboratory of Genetics and Germplasm Innovation of Tropical Special Forest Trees and Ornamental Plants (Ministry of Education), School of Forestry, School of Life Sciences, Hainan University, Haikou, China; ^2^ Haikou Experimental Station, Tropical Crops Genetic Resources Institute, Chinese Academy of Tropical Agricultural Sciences, Key Laboratory of Crop Gene Resources and Germplasm Enhancement in Southern China, Ministry of Agriculture, Key Laboratory of Tropical Crops Germplasm Resources Genetic Improvement and Innovation of Hainan Province, Haikou, China; ^3^ The Engineering Technology Research Center of Tropical Ornamental Plant Germplasm Innovation and Utilization, Danzhou, China

**Keywords:** *Phalaenopsis*-type *Dendrobium*, floral color, anthocyanin biosynthesis, MYB transcription factor, bHLH transcription factor

## Abstract

*Phalaenopsis*-type *Dendrobium* is a popular orchid with good ornamental and market value. Despite their popularity, molecular regulation of anthocyanin biosynthesis during flower development remains poorly understood. In this study, we systematically investigated the regulatory roles of the transcription factors DhMYB2 and DhbHLH1 in anthocyanins biosynthesis. Gene expression analyses indicated that both *DhMYB2* and *DhbHLH1* are specifically expressed in flowers and have similar expression patterns, showing high expression in purple floral tissues with anthocyanin accumulation. Transcriptomic analyses showed 29 differentially expressed genes corresponding to eight enzymes in anthocyanin biosynthesis pathway have similar expression patterns to *DhMYB2* and *DhbHLH1*, with higher expression in the purple lips than the yellow petals and sepals of *Dendrobium* ‘Suriya Gold’. Further gene expression analyses and Pearson correlation matrix analyses of *Dendrobium* hybrid progenies revealed expression profiles of *DhMYB2* and *DhbHLH1* were positively correlated with the structural genes *DhF3’H1*, *DhF3’5’H2*, *DhDFR*, *DhANS*, and *DhGT4*. Yeast one-hybrid and dual‐luciferase reporter assays revealed *DhMYB2* and *DhbHLH1* can bind to promoter regions of *DhF3’H1*, *DhF3’5’H2*, *DhDFR*, *DhANS* and *DhGT4*, suggesting a role as transcriptional activators. These results provide new evidence of the molecular mechanisms of DhMYB2 and DhbHLH1 in anthocyanin biosynthesis in *Phalaenopsis*-type *Dendrobium*.

## Introduction

*Phalaenopsis*-type *Dendrobium* (PD) is a member of the Orchidaceae plant family and is popular around the world because of desirable traits such as vivid floral colors, a variety of forms and a long vase life. The colors and shapes of petals, sepals and lips are the most important ornamental characteristics of PD flowers. These flowers show various patterns and colorations, with anthocyanin-produced purple, peach, or pink colorations frequently observed (Hichri et al., 2011). Although a wide range of flower colors exist in orchids, some species lack specific colors. Blue and orange, for example, are lacking in *Dendrobium* hybrids, and multicolors are still rare (Kuehnle et al., 1997; Yin et al., 2021). In recent years, the breeding of rare floral colors and novel coloration patterns has become an important direction of PD breeding. However, the genome sequence of PD is not yet available, and there are few studies on the regulatory mechanisms between floral color formation and anthocyanin synthesis during flower development. This poor understanding of the regulatory mechanisms of pigmentation limits the ability to create novel floral colors and patterns of PD through genetic engineering technologies.

Anthocyanins, a class of important secondary metabolites in plants belonging to the flavonoids, are water-soluble pigments that give flowers blue, red or purple coloration (Su and Hsu, 2003). Anthocyanins also have important biological roles against various biotic and abiotic stresses. In humans, anthocyanins are reported to have positive effects in preventing cancer and cardiovascular diseases (Khoo et al., 2017). In recent years, more and more studies focus on the molecular mechanism of anthocyanin biosynthesis and composition because of their great value in floral color breeding and their potential health benefits in food.

The anthocyanin biosynthesis pathway (ABP) has been extensively studied in various plants and found to be generally conserved (Tohge et al., 2017; Zhao et al., 2022). The biosynthesis and accumulation of anthocyanins are controlled by various transcription factors (TFs) and ABP structural genes. Initial steps consist of the general phenylpropanoid pathway. L-phenylalanine is metabolized by phenylalanine ammonia-lyase (PAL), then cinnamic acid 4-hydroxylase (C4H), and then 4-coumarate: CoA ligase (4CL) to generate 4-coumaroyl-CoA. 4-coumaroyl-CoA is an important precursor in the biosynthesis of a number of natural products including anthocyanins. The dihydroflavonols are synthesized from 4-coumaroyl-CoA by a series of enzymes such as chalcone synthase (CHS), chalcone isomerase (CHI), flavanone 3-hydroxylase (F3H), flavanone 3'-hydroxylase (F3'H), and flavanone 3'-5'-hydroxylase (F3'5'H). In downstream pathways, the dihydroflavonols are converted into colored anthocyanidins by dihydroflavonol 4-reductase (DFR) and anthocyanidin synthase (ANS). The UDP-glucose anthocyanidin 3-O-glycosyltransferase (UFGT) is required for glycosylation and subsequent stabilization of anthocyanidins. Interestingly, some ABP structural genes show tissue-specific expression pattern during the anthocyanin biosynthesis process. The structural genes are distinguished into two groups: the early biosynthesis genes (EBGs) including *CHS*, *CHI*, and *F3H*; and the late biosynthesis genes (LBGs) including *F3'H*, *F3'5'H*, *DFR*, *ANS*, and *UFGT*. The complete ABP relies on the coordinated expression of EBGs and LBGs (Fang et al., 2019; Yang et al., 2020).

The TFs were also reported to control anthocyanin biosynthesis in plants. Among the TFs involved in ABP, R2R3-MYB, basic helix-loop-helix (bHLH), and WD-repeat (WDR) TFs are the most widely studied. MYB-bHLH-WDR TFs (MBW) usually act as a complex to regulate anthocyanin biosynthesis in plants (Koes et al., 2005; Nemesio-Gorriz et al., 2017; Liu et al., 2018; Zhao et al., 2019). MYB TFs play a critical role in determining the specific target genes and the patterns of color pigmentation in plants (Zhang et al., 2014; Naing and Kim, 2018; Yan et al., 2021). bHLH proteins binding to promoter regions with E-box (CANNTG) or G-box (CACGTG) motifs were found to participate in anthocyanin biosynthesis (Ahmad et al., 2015; Ji et al., 2016; Singh et al., 2021). WDR TFs act as scaffolding molecules, assisting the proper activity of other proteins (Xu et al., 2020; Mackon et al., 2021). Although there are some studies focusing on the mechanism of MYB-bHLH TFs in orchids such as *Oncidium*, *Phalaenopsis*, *Pleione*, and *Cymbidium* (Chiou and Yeh, 2008; Hsu et al., 2015; Hsu et al., 2019; Zhang et al., 2019; Ke et al., 2021; Yang et al., 2021), there are few reports on the molecular mechanisms of TFs in *Dendrobium*. The research on ABP in *Dendrobium* is still limited to structural genes in *D. officinale*, *D. moniliforme*, and *D.* hybrids (Whang et al., 2011; Kriangphan et al., 2015; Yu et al., 2018; Zhan et al., 2020). In our previous study, *DhMYB2* and *DhbHLH1* were cloned from PD and found to be involved in anthocyanin pigmentation in petals. Transient over-expression of *DhMYB2* and *DhbHLH1* resulted in anthocyanin production in white petals (Li et al., 2017). However, the regulatory relationship between TFs and structural genes in anthocyanin synthesis is not clear. The study of the mechanism of TF regulation of anthocyanin synthesis during flower development can provide guidelines for breeding new floral color varieties and improving ornamental quality.

Here, we investigated the spatiotemporal expression profiles of *DhMYB2* and *DhbHLH1* as well as the anthocyanin content in different varieties of PD. In addition, we generated extensive transcriptome data and profiled the correlation between *DhMYB2*/*DhbHLH1* and key structural genes involved in anthocyanin synthesis. We also demonstrated that DhMYB2 and DhbHLH1 can bind to the promoter regions and activate the transcription of ABP structural genes. This study begins to reveal the molecular mechanisms of regulation of structural genes by TFs in anthocyanin biosynthesis of *Phalaenopsis*-type *Dendrobium*.

## Materials and methods

### Plant materials

The *Phalaenopsis*-type *Dendrobium* (PD) were grown under long-day conditions under natural light in Danzhou, Hainan Province, China. The PD cultivars used were *D.* ‘Sonia Hiasakul’, *D.* ‘Udomsri Beauty’, *D.* ‘Burana Stripe’, *D.* ‘Suriya Gold’, and twenty F1 progenies from hybrids *D.* ‘Emma White’ × *D.* ‘Danzhou Ziwei’ (“ED”) or hybrids *D.* ‘Burana Princess’*× D.* ‘Pearl River’ (“BP”). Three floral development stages were defined as described by Li et al. (Li et al., 2017): stage 1, early young bud (~0.5 × 1.0-1.5 cm: width × height); stage 2, mature bud (~1.5 × 1.5-2.0 cm); and stage 3, fully open flower. The sepal, petal, and lip tissues were used for floral color measurement, RT-qPCR, and total anthocyanin content analysis.

### Floral color measurement and total anthocyanin content analysis

The floral color was measured according to previous criteria (Li et al., 2017) using a Chroma Meter (NDK, Japan). Parameters of the CIEL^*^a^*^b^*^ color coordinate including lightness (*L* ^*^, 0 ~ 100, from black to white), chromatic components (a^*^, –100 ~ 100, from green to red), and yellowness (*b* ^*^, –100 ~ 100, from blue to yellow) were measured.

Total anthocyanin content extracted from PD flowers was determined according to previous criteria with appropriate modifications (Li et al., 2017). First, the sepals, petals, and lips of flowers were placed in a 5 mL centrifuge tube. Tissue was mashed with liquid nitrogen, and then a methanol solution with 0.1% hydrochloric acid was added to each tube. Each tube was shaken well and incubated at 4 °C for 24 h. Then, chloroform and 0.1% hydrochloric acid aqueous solution were added to the supernatants after centrifugal separation. The resulting supernatants were analyzed with a spectrophotometer.

### RNA-seq differential gene expression analysis

The purity, concentration and integrity of RNA from *D*. ‘Suriya Gold’ were tested using a NanoDrop 2000 spectrophotometer (Thermo Scientific, Wilmington, DE, USA) to ensure adequate quality for transcriptome sequencing. RNA samples that met the requirements were sent to BioMarker Technologies Co. Ltd. (Beijing, China) for transcriptome sequencing. Nine RNA-seq libraries (sepals, petals and lips with three biological replicates) were constructed and sequenced using the Illumina HiSeq2000 platform. Transcriptome assembly was accomplished using Trinity (Grabherr et al., 2011). Differential expression analysis of three groups was performed using the DESeq R package (1.10.1). The resulting P values were adjusted using the Benjamini and Hochberg’s approach for controlling the false discovery rate. Genes with an adjusted P-value <0.05 found by DESeq were assigned as differentially expressed and were used for Gene Ontology (GO) enrichment and Kyoto Encyclopedia of Genes and Genomes (KEGG) pathway analysis.

### Quantitative real-time PCR

Total RNA was isolated using Total RNA Isolation Kit (FOREGENE, Chengdu, China), and first strand cDNA was synthesized using HiScript II Reverse Transcriptase (Vazyme, Nanjing, China) according to the manufacturer’s instructions. cDNAs were amplified with gene-specific primers which were designed using Primer Premier 5 software. All the primers used in this experiment were listed in **Supplementary Table S3**. RT-qPCR was performed in a 10 μL volume containing 5 μL 2×SYBR^®^Premix Ex Taq ™ (Vazyme, Nanjing, China), 1 μL of the cDNA sample, and 0.2 μM of each gene-specific primer. The PCR conditions were as follows: 95°C for 3 min, 40 cycles of 95°C for 5 s, 60°C for 34 s. Three replicates were used for each sample. The relative expressions of target genes were normalized to the expression of Actin. Reactions were performed on Line-Gene96plus Real-Time PCR System (BIOER TECHNOLOGY, Hangzhou, China).

### Isolation and sequence analysis of promoter regions

Genomic DNA was extracted from PD using the Super Plant Genomic DNA Kit (Tiangen Biotech, Beijing, China). Chromosome walking was performed to isolate the 5' flanking unknown promoter regions of *DhF3'H1*, *DhF3'5'H2*, *DhDFR*, *DhANS*, and *DhGT4* using the Genome Walking Kit (TaKaRa, JAPAN) according to the instructions. TAIL-PCR was used in this experiment with degenerate primers and specific primers, and these primer sequences are listed in **Supplementary Table S4**. Subsequently, *cis*-acting elements of promoter regions were predicted using the online tools PLACE (https://www.dna.affrc.go.jp/PLACE/action=newplace) and PlantCARE (http://bioinformatics.psb.ugent.be/webtools/plantcare/html).

### Yeast assays

For yeast one-hybrid assays, the CDS of *DhMYB2* and *DhbHLH1* were cloned into the GAL4 transcriptional activation pGADT7 vector (Clontech, USA). DNA fragments corresponding to the promoters of the target genes (*DhF3'H1*, *DhF3'5'H2, DhDFR*, *DhANS*, *DhGT4*) were separately inserted into the pABAi plasmid (Invitrogen, USA). These constructs were then transformed into the yeast strain Y1Hgold. The p53-ABAi was co-transformed with pGADT7-53 as a positive control (Invitrogen, USA). Yeast one-hybrid assays were performed following the manufacturer’s instructions (Invitrogen, USA). The transformation clones were selected in synthetic dropout (SD) medium without leucine and uracil which contain AbA at different gradients for positive screening.

### Dual luciferase transcriptional activity assays

The TFs were cloned into the expression vector pGreen 62-SK for dual luciferase assays. The upstream 1 to 2-kb region of target genes were inserted into a luciferase reporter. Then, the constructs were transformed into *Agrobacterium tumefaciens* strain GV3101 and co-infiltrated into leaves of *N*. *benthamiana* for 48 hours. The Dual Luciferase Reporter Assay System (Promega, USA) was used to measure the luciferase activity by Tecan Infinite M200 (Tecan, Switzerland).

### Statistical analyses and correlation analyses

Statistical tests, significance analyses, and correlation analyses were performed using SPSS 26.0 and Origin 2021.

## Results

### *DhMYB2* and *DhbHLH1* are specifically expressed in flowers associated with anthocyanin biosynthesis

**Figure 1:**
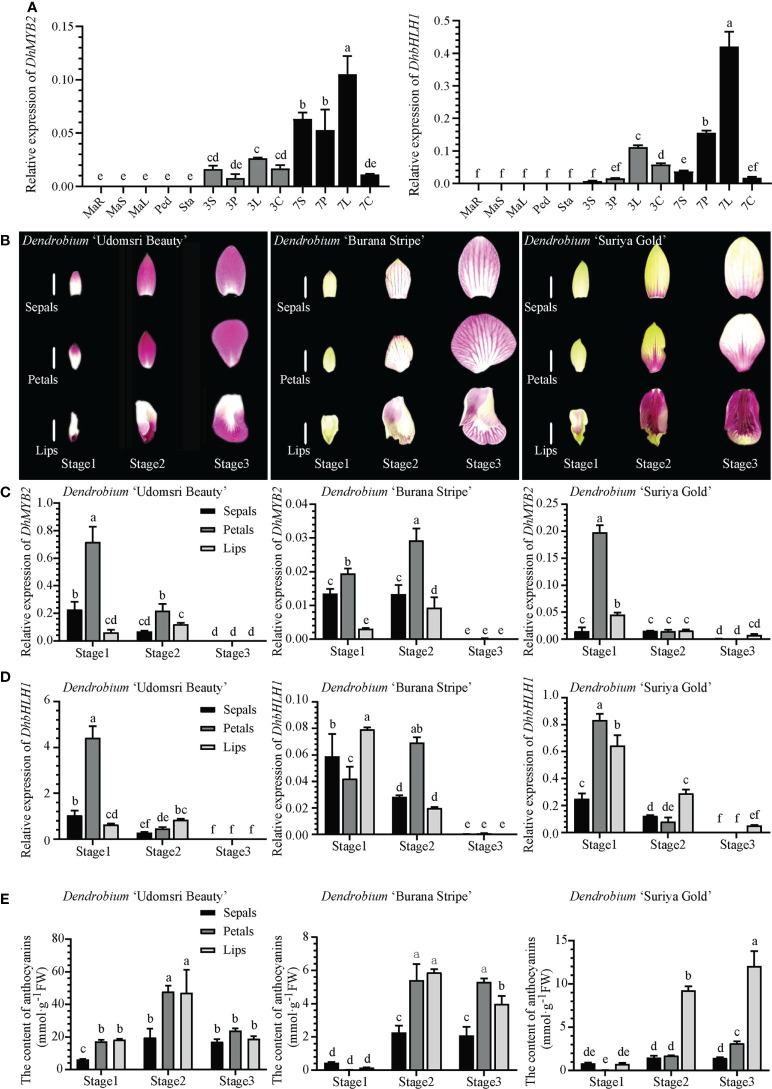
Expression patterns of *DhMYB2* and *DhbHLH1* in different *Dendrobium* cultivars. **(A)** Tissue specific expression patterns of *DhMYB2* and *DhbHLH1* in *D. ‘*Sonia Hiasakul’. *MaR*, Mature roots; *MaS*, Mature stems; *MaL*, Mature leaves; *Ped*, Pedicels; *Sta*, Stalks; *3S*, sepals of 3 mm buds; *3P*, petals of 3 mm buds; *3L*, lips of 3 mm buds; *3C*, stamens of 3 mm buds; *7S*, sepals of 7 mm buds; *7P*, petals of 7 mm buds; *7L*, lips of 7 mm buds; *7C*, stamens of 7 mm buds. **(B)** Floral color phenotypes in three developmental stages of *D. ‘*Udomsri Beauty’, *D. ‘*Burana Stripe’ and *D. ‘*Suriya Gold’, Bar=1.0 cm. RT-qPCR expression analyses of *DhMYB2* **(C)** and *DhbHLH1* **(D)**, and anthocyanin content **(E)** of different floral tissues of *D. ‘*Udomsri Beauty’, *D. ‘*Burana Stripe’ and *D. ‘*Suriya Gold’ from three developmental stages. RT-qPCR expression values are expressed as mean -dCt (Ct reference - Ct target) ± SEM from three biological replicates using *actin* as an internal control for normalization. Different letters indicate significant differences (*p* < 0.05, Duncan’s multiple range tests).

In order to understand the tissue expression patterns of *DhMYB2* and *DhbHLH1*, RT-qPCR was performed on various tissues from *D.* ‘Sonia Hiasakul’. *DhMYB2* and *DhbHLH1* are specifically expressed in flowers while unexpressed in other vegetative tissues. Expression of both *DhMYB2* and *DhbHLH1* increase significantly during flower development in sepals, petals and lips (**Figure 1A**). Subsequently, cultivars with different floral color phenotypes (*D.* ‘Udomsri Beauty’, *D.* ‘Burana Stripe’ and *D.* ‘Suriya Gold’) were used to further study the expression patterns of *DhMYB2* and *DhbHLH1* (**Figure 1B**). Dissected sepals, petals, and lips from bracts in three development stages were used to examine the temporal and spatial expression patterns. In *D.* ‘Udomsri Beauty’, both *DhMYB2* and *DhbHLH1* are highly expressed in sepals and petals at stage 1 and have reduced expression in later development (**Figures 1C, D**). Meanwhile, *DhMYB2* and *DhbHLH1* have similar expression patterns in *D.* ‘Burana Stripe’ and *D.* ‘Suriya Gold’ but lower expression levels than observed in *D.* ‘Udomsri Beauty’. Interestingly, *DhbHLH1* had high expression levels in lips of *D.* ‘Udomsri Beauty’ and *D.* ‘Suriya Gold’, both of which display purple red coloration.

To explore the relationship between anthocyanins and expression of *DhMYB2* and *DhbHLH1* TFs, we determined the anthocyanin content in the flowers of these three PD cultivars. As shown in **Figure 1E**, the anthocyanin content is highest in *D.* ‘Udomsri Beauty’. Anthocyanin content is highest in stage 2 and is maintained in stage 3. Meanwhile, the lips of *D.* ‘Suriya Gold’ which display deep purple red coloration, have increased anthocyanin content as compared to their yellow petals and sepals, consistent with *D.* ‘Udomsri Beauty’. In general, tissues with high TF expression levels accumulate anthocyanins later in development. For example, the petals of *D.* ‘Udomsri Beauty’ show high expression of both TFs in stage 1 and then peak anthocyanin content in stage 2 petals. Similarly, *D.* ‘Burana Stripe’ shows moderate levels of *DhbHLH1* in all three tissues in stage 1 and elevated anthocyanin content in all three tissues in stages 2 and 3. These results suggest the possibility that *DhMYB2* and *DhbHLH1* may regulate the accumulation of anthocyanins.

### Identification of differentially expressed TFs and ABP genes in *Dendrobium* floral tissues of various colors *via* transcriptome analyses

To investigate the potential regulatory roles of DhMYB2 and DhbHLH1 TFs during anthocyanin accumulation in *Dendrobium*, we performed RNA-Seq transcriptome analyses of different floral tissues (petals, sepals and lips) of *D.* ‘Suriya Gold’ with various color phenotypes. After removing low-quality reads, more than 19 million clean reads were obtained from each library, with greater than 90% of bases meeting high-quality Q20 or Q30 scoring thresholds (**Table 1**). 49,938 unigenes were obtained after assembly, with N50 length of 2,837 bp. 38,833 unigenes were annotated as functional genes by BLASTX (e ≤ 1.00 × 10^−5^). (**Figure S1** and **Table S1**). We performed further functional annotation of unigenes using COG and KOG databases, with major functional classifications including post-translational modification, protein transport, and chaperone associated proteins. Statistics are presented in **Figure S2**.

**Table 1:** Evaluation statistics of sample sequencing data.

Sample	Sample ID	Read Sum	GC (%)	Q20 (%)	Q30 (%)
GS1	T24	29051366	46.77	97.94	94.31
GS2	T25	26923363	47.08	98.15	94.79
GS3	T26	28314147	46.64	98.10	94.70
GP1	T18	20178877	46.75	98.36	95.34
GP2	T19	21622216	46.80	98.07	94.70
GP3	T20	22228953	47.01	98.21	94.93
GL1	T21	22034196	47.07	97.94	94.53
GL2	T22	21315317	47.52	98.20	94.95
GL3	T23	19784284	47.30	97.95	94.54

Q30 (%), percentage of bases with Clean Data mass value greater than or equal to 30.

**Figure 2:**
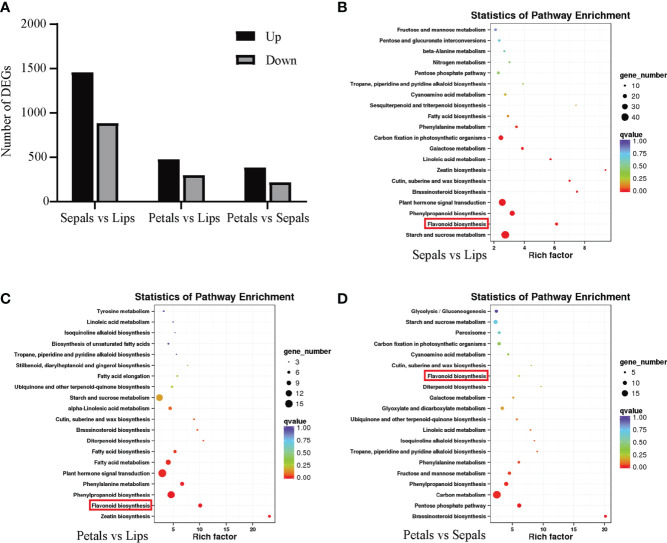
KEGG pathway enrichment analyses of DEGs of different floral tissues of *D. ‘*Suriya Gold’. **(A)** Number of DEGs upregulated and downregulated in each comparison. KEGG pathway enrichment scatter plots of DEGs in each comparison: Sepals vs Lips **(B)**, Petals vs Lips **(C)** and Petals vs Sepals **(D)**. Top 20 significant pathways involving DEGs are included, with the flavonoid metabolism pathway highlighted with red rectangles.

To identify differentially expressed genes (DEGs) between tissues, we compared the FPKM values of each DEG in different floral tissues. There are 2340 DEGs between sepals and lips, 774 DEGs between petals and lips, and 599 DEGs between petals and sepals. Among the above respective comparisons, 1458, 476, and 383 DEGs are upregulated, and 882, 298, and 383 DEGs are downregulated (**Figure 2A**). The DEGs were used to identify the top 20 enriched metabolic pathways through the KEGG pathway database. Notably, the flavonoid biosynthesis pathway is enriched in DEGs of all three comparisons (**Figures 2B-D**).

**Figure 3:**
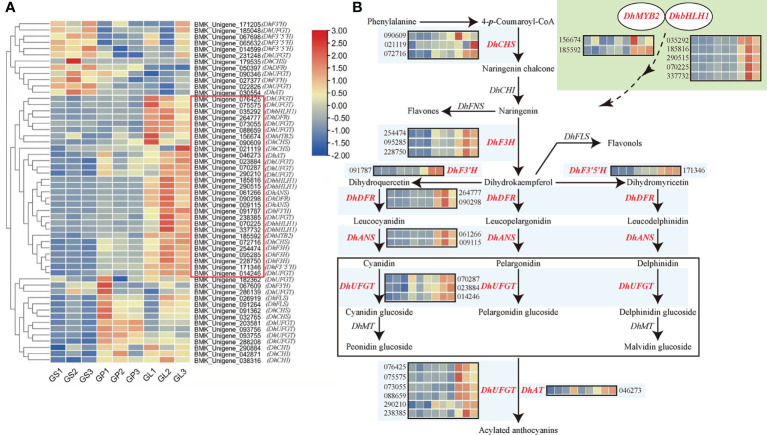
Clustered heatmap showing the expression patterns of transcription factor genes and flavonoid biosynthesis related DEGs in different floral tissues of *D. ‘*Suriya Gold’. **(A)** Cluster analysis of *DhMYB2* and *DhbHLH1* as well as differentially expressed genes involved in the flavonoid and anthocyanin biosynthesis pathway. The log_2_ ratio values of the expression in each comparison of DEGs were used for cluster analysis with the R heatmap package. The color changes from blue (low level) to red (high level) represent the log2FPKM values measured from different floral tissues of *D. ‘*Suriya Gold’. The red rectangle highlights the unigenes with similar expression profiles as *DhMYB2* and *DhbHLH1*. *GS 1-3*, *GP 1-3* and *GL 1-3* represent sepals, petals, and lips of *D. ‘*Suriya Gold’ from three biological replicates, respectively. **(B)** Schematic diagram of ABP in *Phalaenopsis*-type *Dendrobium*, illustrating the eight highlighted genes with similar expression profiles as *DhMYB2* and *DhbHLH1*.

Subsequently, we focused on an expression profile analysis of *DhMYB2*, *DhbHLH1*, and anthocyanin biosynthesis related genes. Two DEGs correspond to *DhMYB2*, five DEGs correspond to *DhbHLH1*, and 48 DEGs correspond to 12 anthocyanin biosynthetic genes that relate to flavonoid biosynthesis pathway (KO00941) or ABP (KO00942). These were selected for further expression cluster analyses in different floral tissues (**Table 2**). Interestingly, 29 of the 48 anthocyanin biosynthesis DEGs have similar expression profiles as *DhMYB2* and *DhbHLH1*, which are highly expressed in the purple red lips of *D.* ‘Suriya Gold’, while they show low expression levels in yellow petals and sepals (**Figure 3A**). These DEGs correspond to anthocyanin biosynthesis genes: *DhCHS, DhF3H, DhF3'H, DhF3'5'H, DhDFR, DhANS, DhUFGT, and DhAT* (**Figure 3B**). These results suggest that *DhMYB2* and *DhbHLH1* may be involved in molecular regulation of select anthocyanin biosynthesis genes.

**Table 2:** Differentially expressed genes (DEGs) information related to anthocyanin biosynthesis pathway.

Gene name	Encoding Protein	DEGs number
*DhCHS*	Chalcone synthase	091362;072716;032765;179535;090609;021119
*DhCHI*	Chalcone isomerase	042871;290884;038316
*DhF3H*	flavanone 3-hydroxylase	254474;095285;228750
*DhFLS*	Flavonol synthase	026919;091264
*DhF3'H*	Flavonoid 3'-hydroxylase	067609;091787;027377;171205
*DhF3'5'H*	Flavonoid 3',5'-hydroxylase	171346;067698;065632;014599
*DhDFR*	Dihydroflavonol 4-reductase	050397;264777;090298
*DhANS*	Anthocyanidin synthase	061266;009115
*DhUFGT*	UDP-glucose anthocyanidin 3-O-glycosyltransferase	182362;070287;185048;022826;014246;090346;286139; 231248;023884;290210;073055;238385;076425;088659; 075575;093755;203581;288208;093756
*DhAT*	Anthocyanin 3-O-glucoside-6'-O-malonyltransferase	046273; 030554
*DhMYB2*	MYB2	156674;185592
*DhbHLH1*	bHLH1	035292;070225;185816;290515;337732

### Correlations between flower color, anthocyanin content, and expression of TFs and select ABP genes

**Figure 4:**
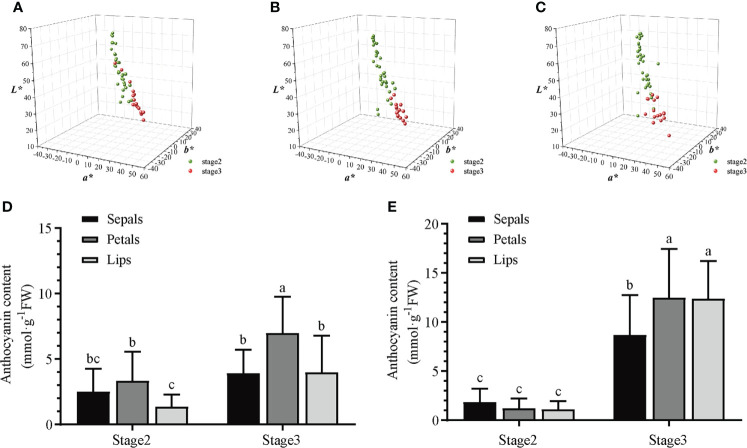
Analyses of color and total anthocyanin content in different floral tissues of two *Dendrobium* hybrid families during flower development. Colorimetric analyses of sepals **(A)**, petals **(B)** and lips **(C)** of “ED” hybrid progenies at different developmental stages in three-dimensional color space using the CIE L^*^a^*^b^*^ system. Analyses of anthocyanin contents (mean ± SEM) in different floral tissues of “ED” hybrid progenies **(D)** or **“**BP” hybrid progenies **(E)** during flower development. Twenty F1 progenies of each hybrid family were used for analyses. Different letters indicate significant differences (*p* < 0.05, Duncan’s multiple range tests).

To further explore the relationship between coloration, anthocyanin content, and gene expression, twenty F1 progenies from two *Dendrobium* hybrid families (“ED” and “BP”) were selected and assayed for coloration and anthocyanin content in the sepals, petals and lips of the bud (stage 2) and early flowering (stage 3). Using a CIE L^*^a^*^b^*^ color coordinate system, floral colors in “ED” hybrid progenies have *L* ^*^ values from 35 to 60, a^*^ values ranging from –10 to 50, and b^*^ values from –25 to 20. Meanwhile, the *L* ^*^ values are lowest in lips at stage 3, showing darker coloration than sepals and petals (**Figures 4A-C**). Similar color change patterns are observed in “BP” hybrid progenies (**Figure S3**). Anthocyanin contents increase from stage 2 to stage 3 in both *Dendrobium* hybrid families (**Figures 4D, E**). Thus, coloration changes and anthocyanin contents are correlated in different floral tissues and stages (**Table S2A, B**).

**Figure 5:**
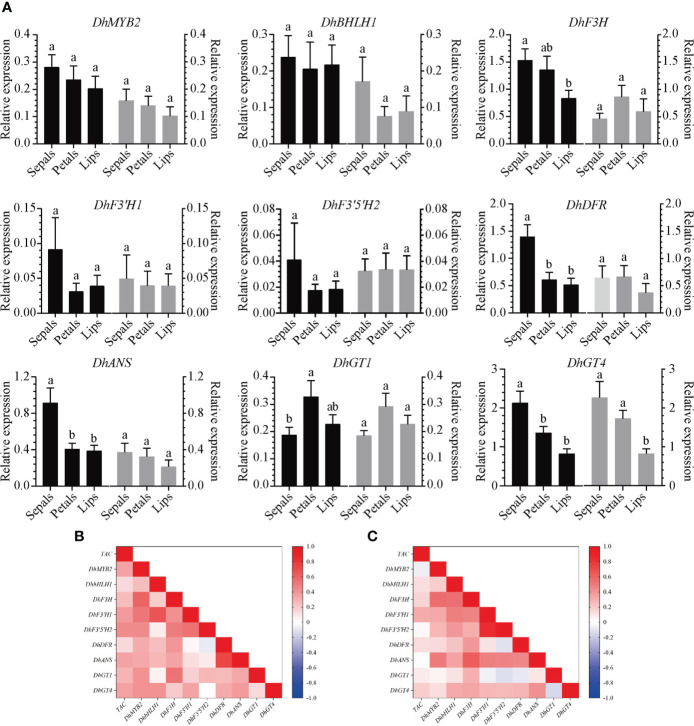
Correlation analyses between the expression of anthocyanin biosynthesis related genes and anthocyanin content in different floral tissues of two *Dendrobium* hybrid families. **(A)**. Expression profiles of anthocyanin biosynthesis related genes in sepals, petals and lips of *Dendrobium* hybrid progenies. The black columns represent “BP” hybrid progenies while the gray columns represent “ED” hybrid progenies. Values are expressed as mean -dCt (Ct reference - Ct target) ± SEM using *actin* as an internal control for normalization. Twenty F1 progenies of each hybrid family were sampled. Different letters indicate significant differences (*p* < 0.05, Duncan’s multiple range tests). Comprehensive correlation matrix analyses between anthocyanin biosynthesis related genes and total anthocyanin content (TAC) in combined sepals, petals and lips of F1 progenies from “ED” **(B)** and “BP” **(C)**.

Anthocyanin biosynthesis related genes (*DhF3H*, *DhF3′H1*, *DhF3′5′H2*, *DhDFR*, *DhANS*, *DhGT1*, and *DhGT4*) with similar expression profiles as *DhMYB2* and *DhbHLH1* were used for further analyses in *Dendrobium* hybrid progenies. Relative expression of these genes (including *DhMYB* and *DhbHLH1*) was determined *via* RT-qPCR (**Figure 5A**). Using Pearson correlation analyses between gene expression and anthocyanin content, we found a positive correlation in “ED” hybrid progenies between total anthocyanin content and expression of *DhMYB2*, *DhF3'H1*, *DhF3'5'H2*, *DhANS*, and *DhGT4* (**Figures 5B** and **S4**). In “BP” hybrid progenies, total anthocyanin content is positively correlated with *DhF3'H1* expression (**Figures 5C** and **S4**). When comparing expression of TFs and ABP structural genes in “ED” hybrid progenies using Pearson comprehensive correlation analyses, we found positive correlation of *DhMYB2* expression with expression of *DhF3H*, *DhF3'H1*, *DhF3'H2*, *DhF3'5'H2*, *DhDFR*, *DhANS*, *DhGT1*, and *DhGT4* and positive correlation of *DhbHLH1* expression with expression of *DhF3’H1* and *DhGT4.* In “BP” hybrid progenies, *DhMYB2* expression correlates positively with *DhF3H*, *DhF3'H1*, *DhF3'5'H2*, and *DhANS* expression, while *DhbHLH1* expression positively correlates with *DhF3H*, *DhF3'H1*, *DhF3'5'H2*, *DhANS*, and *DhGT4* expression (**Figures 5A, B** and **Figure S4**). These correlations are consistent with regulation of anthocyanin synthesis and accumulation by these TFs and ABP structural genes.

### Interactions between TFs and the promoters of ABP structural genes

**Figure 6:**
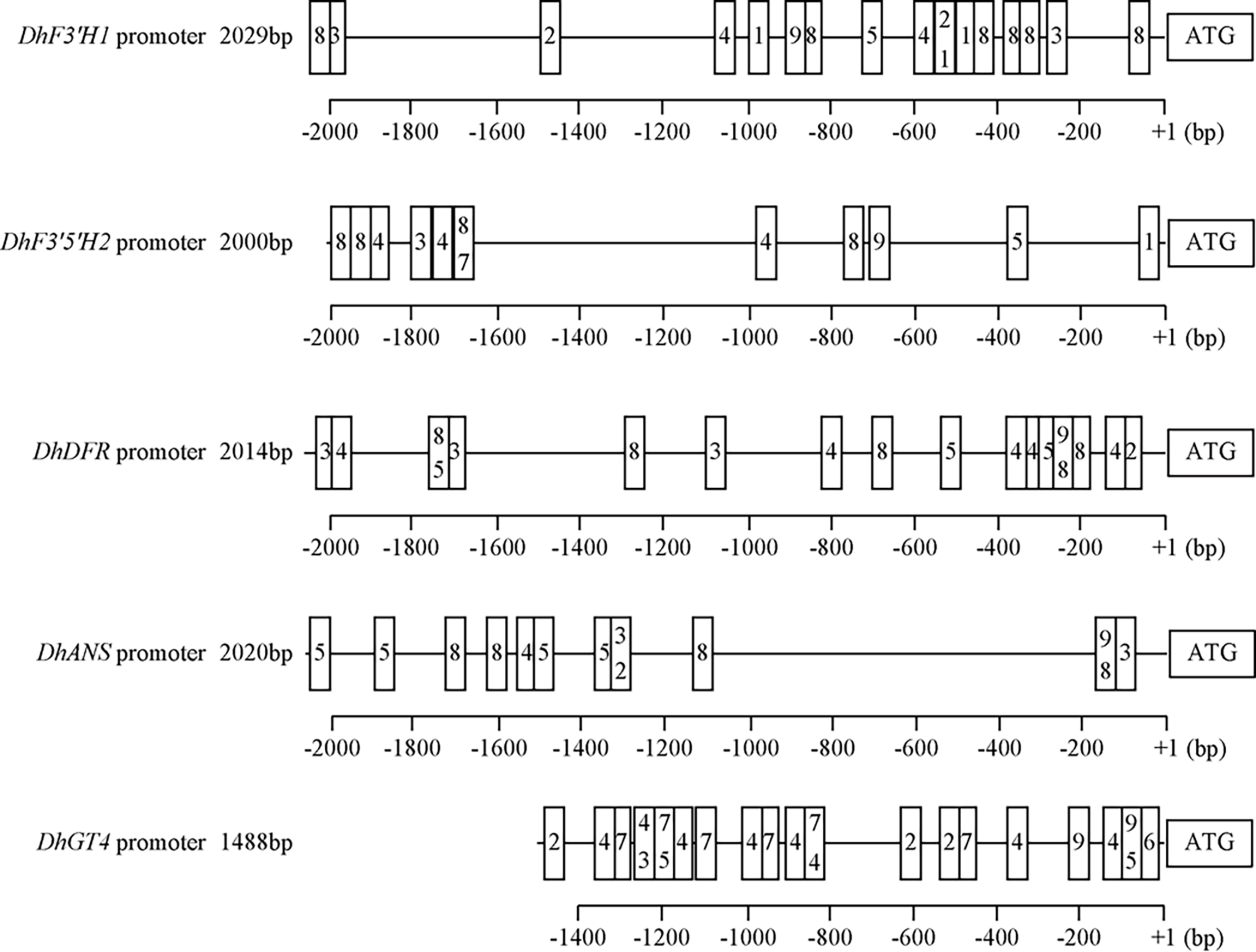
Schematic diagram of MYB and bHLH binding-site motifs predicted in the promoters of ABP structural genes. Possible *cis*-regulatory elements recognized by MYB or bHLH were predicted using the promoter regions of *DhF3’H1*, *DhF3’5’H2, DhDFR*, *DhANS*, and *DhGT4*, respectively. Numbers in boxes indicate potential MYB binding-site motifs: 1, MRE; 2, MYBPLANT; 3, MYBPZM; 4, MYBCORE; 5, MYB1AT; 6, MBSI; 7, MBS; and potential bHLH binding-site motifs: 8, E-box; 9, G-box.

**Figure 7:**
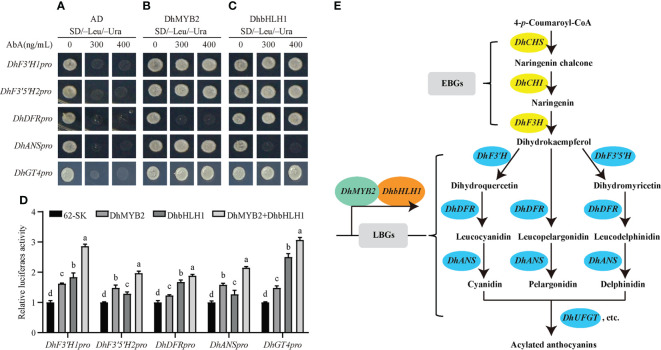
Yeast one-hybrid (Y1H) and dual‐luciferase reporter assays showing the association of *DhMYB2* and *DhbHLH1* with promoters of ABP structural genes. The Y1HGold yeast stains harboring the promoter reporter were transformed with empty plasmid as negative control **(A)**, effector *DhMYB2* **(B)**, or effector *DhbHLH1* **(C)**, respectively. Interaction was detected based on the ability of transformed stains to grow on SD medium lacking Leu and Ura in the presence of 300 or 400 ng/mL AbA. **(D)** Luciferase activity in tobacco leaves was detected 3 days after infiltration. Firefly luciferase (LUC) activities were normalized against Renilla luciferase (REN) activities. Graphs are presented as mean ± SEM from three biological replicates. Different letters indicate significant differences (*p* < 0.05, Duncan’s multiple range tests). **(E)** Schematic representation of the suggested role of DhMYB2 and DhbHLH1 in ABP. Metabolic enzymes involved in the pathway are indicated. DhMYB2 and DhbHLH1 binding to promoter of LBGs act as activators in ABP.

To further explore TF‐mediated regulation of anthocyanin synthesis and accumulation through ABP structural genes, we selected five structural genes with significant positive correlations with the TFs *DhMYB2* or *DhbHLH1* (*DhF3'H1*, *DhF3'5'H2*, *DhDFR*, *DhANS* and *DhGT4*) to study in detail. First, we isolated the 2029 bp promoter region of *DhF3’H1*, 2000 bp of *DhF3’5’H2*, 2014 bp of *DhDFR*, 2020 bp of *DhANS*, and the 1488 bp *DhGT4* promoter region using the genome walking technique. Subsequently, we surveyed these promoter regions for putative MYB and bHLH binding sites. Multiple putative MYB and bHLH recognition binding elements were identified in the promoter regions of five structural genes (**Figure 6** and **Table S3A-E**), suggesting that the five genes might be directly regulated by MYB and bHLH. Finally, to demonstrate DNA binding activity of DhMYB2 and DhbHLH1 in these target regions, yeast one-hybrid and luciferase assays were performed. The Y1H showed that DhMYB2 directly binds to promoters of *DhF3'H1*, *DhF3'5'H2*, *DhANS*, and *DhGT4*, while DhbHLH1 binds to promoters of *DhF3'H1, DhF3'5'H2, DhDFR*, and *DhGT4* (**Figures 7A-C**). Consistent with a role of these TFs as direct transcriptional activators of ABP structural genes, dual‐luciferase reporter assays showed that all five structural genes can be activated by DhMYB2 and DhbHLH1 individually by 1.5 to 2.5 fold. Interestingly, co-transformation of *DhMYB2* and *DhbHLH1* together with structural genes promoters significantly elevates luciferase activity as compared to each TF alone (**Figure 7D**).

## Discussion

*Phalaenopsis*-type *Dendrobium* is an important ornamental tropical orchid, with diverse pigmentation patterns and longevity making it popular worldwide. Breeding of rare floral colors or novel coloration patterns would further increase the value of PD. Unfortunately, few studies have focused on the pigment compositions and molecular mechanisms of PD flower development. Our group previously identified two TFs (DhMYB2 and DhbHLH1) as part of complexes involved in anthocyanin synthesis in PD (Li et al., 2017). Here, we systematically investigated the regulatory roles of DhMYB2 and DhbHLH1 in the accumulation of anthocyanins that underlie floral colors and further studied the molecular mechanisms of these two TFs as transcriptional activators of key structural genes in the anthocyanin biosynthesis pathway.

### The expression patterns of *DhMYB2* and *DhbHLH1* are correlated with anthocyanin accumulation and pigmentation

Anthocyanin biosynthesis is mainly regulated by TFs and structural genes. MYB and bHLH TFs, such as PeMYB11 in *Phalaenopsis* Orchids and CmbHLH2 and CmMYB6 in *Chrysanthemum*, have been identified to be the most important TFs in the ABP in plants (Hsu et al., 2019; Lim et al., 2021). In this study, *DhMYB2* and *DhbHLH1* were found to be specifically expressed in flowers of PD (**Figure 1A**), suggesting that they might play an important role in anthocyanin accumulation in the flower. Similar flower-specific expression of the TFs *B-peru* and *mPAP1* have been found to enhance floral color in tobacco (Kim et al., 2018). Flower-specific expression of *PpMYB15* and *PpMYBF1* in *Prunus persica* and R2R3-MYB *EOBII* in Petunia have also been reported (Czemmel et al., 2009; Cao et al., 2019). Moreover, we found *DhMYB2* and *DhbHLH1* were highly expressed in the floral tissues with purple color. Interestingly, high expression of the TFs in tissues was always accompanied by subsequent anthocyanin accumulation. We speculate that the regulation of TFs is upstream of the anthocyanin biosynthetic pathway in PD, and that the synthesis of anthocyanins act as the products of metabolites, while lags behind the expression of genes. The spatiotemporal expression patterns of *DhMYB2* and *DhbHLH1* are consistent with the changes in anthocyanin accumulation and purple color formation in PD (**Figure 1B-E**). TFs have been previously shown to positively correlate with anthocyanin synthesis and pigmentation. For example, *McMYB10* expression is significantly correlated with anthocyanin synthesis and red pigmentation in crabapple (Tian et al., 2015). Nuraini et al. (Nuraini et al., 2020) also reported the spatiotemporal expression profiles of *MiMYB1* and *MibHLH1* are correlated with anthocyanin accumulation profiles in *Matthiola incana*. Consequently, DhMYB2 and DhbHLH1 might be key TFs involved in anthocyanin accumulation and purple floral color formation in PD.

### The expression profiles of *DhMYB2* and *DhbHLH1* positively correlate with the LBGs related to pigmentation

Transcriptomic analyses have been performed to identify critical genes for anthocyanin accumulation (Jiang et al., 2020; Zhang et al., 2020; Zhou et al., 2020). In our study, 29 DEGs corresponding to eight enzymes of ABP have expression patterns similar to *DhMYB2* and *DhbHLH1*, with high expression found only in purple lips (**Figure 3**). These ABP structural genes were considered as candidate regulatory targets of the two TFs in the process of anthocyanin accumulation. In order to investigate the relationship between TFs and candidate structural genes during anthocyanin accumulation in PD, twenty F1 progenies from two hybrid families were selected for systematic studies. Interestingly, the expression profiles of *DhF3'H1*, *DhF3'5'H2*, *DhDFR*, *DhANS*, and *DhGT4*, all LBGs, are significantly positively correlated with those of *DhMYB2* or *DhbHLH1*, and significant correlations were also found between some of these genes and total anthocyanin content in the two hybrid families (**Figure 5**). Anthocyanin biosynthesis depends on expression of both EBGs and LBGs (Jiang et al., 2020; Tang et al., 2020; Li et al., 2021a). Regulation of anthocyanin accumulation by MYB or bHLH-mediated activation of LBGs has previously been reported in many plants (Chiu et al., 2010; Zhou et al., 2014; Sun et al., 2016; Rameneni et al., 2020). In addition, it has been widely reported that activation of LBGs is associated with red pigmentation. For example, *F3'5'H* is involved in purple colored pigmentation in *D. moniliforme* (Whang et al., 2011), and similar results have been reported with *ChF3’H* and *ChANS* in *Cymbidium orchid*, *PlANS* and *PlUFGT* in *Pleione* ‘limprichtii’, and *PeUFGT3* in *Phalaenopsis* (Chen et al., 2011; Wang et al., 2014; Zhang et al., 2020). Taken together, our results indicate that *DhF3'H1*, *DhF3'5'H2*, *DhDFR*, *DhANS*, and *DhGT4* might be the downstream target genes regulated by DhMYB2 or DhbHLH1 in ABP and floral color formation.

### Direct interactions between TFs and structural genes involved in anthocyanin biosynthesis

It is widely reported that MYB-bHLH usually acts as a complex to regulate anthocyanin biosynthesis in plants (Baudry et al., 2004; Wang et al., 2020; Kim et al., 2021). MYB can not only play a regulatory role alone, but also interacts with bHLH to regulate anthocyanin biosynthesis and color formation in plants (Espley et al., 2007; Yuan et al., 2013; Albert et al., 2021). MYB and bHLH can directly bind to *cis*-acting elements of gene promotors to regulate the expression of ABP structural genes and subsequent pigment formation. Shen et al. (Shen et al., 2014) previously demonstrated that PacMYBA interacts with bHLH to activate the promoters of *PacDFR*, *PacANS*, and *PacUFGT* and regulates pigmentation in sweet cherry. Besides acting as a complex, MYB and bHLH can also regulate anthocyanin biosynthesis alone (Xiang et al., 2015; Qi et al., 2020). MYB binding elements involved in flavonoid biosynthesis have been identified in many reports, including AGMOTIFNTMYB2, MYB26PS, MYBPZM, MYB core, and MYBPLANT (Singh et al., 2015; Yan et al., 2021). Likewise, E-box *cis*-acting elements are the most common binding targets of bHLH, of which G-box is the most common type (Li et al., 2021b; Qian et al., 2021). In this study, several putative *cis*-elements recognized by MYB or bHLH were identified in the promoter regions of *DhF3'H1*, *DhF3'5'H2, DhDFR*, *DhANS*, and *DhGT4* (**Figure 6**).

Y1H and dual‐luciferase reporter assays were used to study the regulatory mechanisms of DhMYB2 and DhbHLH1. We found *DhMYB1* can positively regulate the expression of *DhF3'H1*, *DhF3'5'H2*, *DhANS*, and *DhGT4* by directly binding to their promoters. Similar results were found in the regulation of *DhF3'H1*, *DhF3'5'H2*, *DhDFR*, and *DhGT4* by *DhbHLH1*. Interestingly, the DhMYB2-DhbHLH1 complex shows stronger activation ability of these structural genes than either TF alone (**Figures 7A-D**), consistent with previous studies in other plants. The combination of *CmMYB6* and *MrbHLH1* activates the *CmDFR* promoter 4-fold higher than that of *CmMYB6* alone (Liu et al., 2015), and similar results have been reported in tobacco (Chen et al., 2017). Direct interactions of MYB or bHLH alone with promoters of structural genes has not been previously shown. Rather, they regulate ABP structural genes by forming MBW protein complexes (Li et al., 2021b; Yan et al., 2021). Similarly, we found DhMYB2 and DhbHLH1 alone are not sufficient to bind and activate expression of *DhDFR* and *DhANS*, respectively, in PD. Although MYB and bHLH recognition sites were identified in both *DhDFR* and *DhANS* promoters, direct binding *via* Y1H was not observed. Additional factors such as the surrounding sequences of the *cis*-elements or variation in particular amino acid domains of TFs may negatively affect binding affinities of TFs (Liu et al., 2016). Somewhat surprisingly, modest luciferase activation of all ABP structural genes was observed for each of these TFs alone in the exogenous *N. benthamiana* system. Exogenous overexpressed TFs can play roles by interacting with endogenous proteins (Lim et al., 2020). Although DhMYB2 alone cannot bind directly to the promoter of *DhDFR* in the Y1H assay, transient overexpression of *DhMYB2* alone in *N. benthamiana* is enough to induce *DhDFR* expression, suggesting that DhMYB2 can play a regulatory role interacting with endogenous TFs in *N. benthamiana*. Meanwhile, *DhbHLH1* might induce the expression of *DhANS* in *N. benthamiana via* a similar mechanism. Our results suggest that DhMYB2 and DhbHLH1 are key TFs regulating anthocyanin biosynthesis and purple pigmentation in PD by direct activation of LBGs (**Figure 7E**).

## Conclusion

In this study, we characterized flower-specific expression of the TF genes *DhMYB2* and *DhbHLH1* in *Phalaenopsis*-type *Dendrobium* and investigated their spatiotemporal expression profiles in PD cultivars with different floral color phenotypes. *DhMYB2* and *DhbHLH1* have similar expression patterns, with high levels observed in floral tissues with high anthocyanin accumulation profiles and purple coloration. In addition, expression levels of *DhMYB2* and *DhbHLH1* are positively correlated with expression of LBGs (*DhF3'H1*, *DhF3'5'H2*, *DhDFR*, *DhANS*, and *DhGT4*) and anthocyanin accumulation according to transcriptomic data of *D.* ‘Suriya Gold’ and Pearson correlation matrix analyses of *Dendrobium* hybrid progenies. Furthermore, Y1H and dual‐luciferase reporter assays show that *DhMYB2* and *DhbHLH1* can activate expression of LBGs through direct binding to their promoters. Altogether, our results provide fresh insights on the mechanisms of DhMYB2 and DhbHLH1 regulation of ABP in PD.

## Data availability statement

The data presented in the study are deposited in the NCBI repository, accession number PRJNA880415.

## Author contributions

YW, CL, JY, and XS conceived the study. YW conducted the main experiment, analyzed the data, and wrote the manuscript. WZ, ZL, and HY participated in the performance of the experiments. CL revised the manuscript. All authors contributed to the article and approved the submitted version.

## Funding

This research was funded by the National Key R&D Program of China (grant no. 2018YFD1000405), the Hainan Provincial Natural Science Foundation of China (No. 320QN186), the Central Public-interest Scientific Institution Basal Research Fund (No. 1630032022002), the earmarked fund for CARS-23-G60.

## Acknowledgments

We sincerely thank Prof. Jian Wang from College of Forestry Hainan University and Dr. Shunjiao Lu from Tropical Crops Genetic Resources Institute, The Chinese Academy of Tropical Agricultural Sciences for advice to the article.

## Conflict of interest

The authors declare that the research was conducted in the absence of any commercial or financial relationships that could be construed as a potential conflict of interest.

## Publisher’s note

All claims expressed in this article are solely those of the authors and do not necessarily represent those of their affiliated organizations, or those of the publisher, the editors and the reviewers. Any product that may be evaluated in this article, or claim that may be made by its manufacturer, is not guaranteed or endorsed by the publisher.

## Supplementary material

Supplementary Figure 1 Length distribution of unigenes in *D.* ‘Suriya Gold’. Length distribution of unigenes identified in transcriptome assembly of *D.* ‘Suriya Gold’. Total number (49,938), Total length (96,387,028 nt), Mean length (1930.13 nt), N50 length (2837 nt). N50 is defined as the length of the longest unigene such that all unigenes of at least N50 length contain greater than 50% of all bases in the transcriptome assembly.

Supplementary Figure 2 COG and KOG functional classifications of unigenes in *D.* ‘Suriya Gold’. (A) COG (Cluster of orthologous groups) functional classifications of unigenes. (B) KOG (Eukaryotic Ortholog Groups) functional classifications of unigenes. The x-axes represent COG or KOG categories, and the y-axes represent the number of unigenes annotated.

Supplementary Figure 3 Analyses of color in different floral tissues of “BP” hybrid progenies during flower development. Colorimetric analyses of sepals **(A**), petals **(B)** and lips **(C)** of “BP” hybrid progenies at different developmental stages in three-dimensional color space using the CIE *L^*^a^*^b^*^* system.

Supplementary Figure 4 Correlation analyses between anthocyanin biosynthesis related genes and anthocyanin content in different floral tissues of two *Dendrobium* hybrid families. Correlation matrix analyses between anthocyanin biosynthesis related genes and total anthocyanin content (TAC) in different floral tissues (sepals, petals and lips) in hybrid progenies from “ED” or “BP”, respectively. Twenty F1 progenies of each hybrid family were sampled.
