# Supplementary figures and images for: DhMYB2 and DhbHLH1 regulates anthocyanin accumulation via activation of late biosynthesis genes in Phalaenopsis-type Dendrobium
Source: Front Plant Sci. 2022 Nov 15;13:1046134. doi: 10.3389/fpls.2022.1046134 (PMC9705975; doi:10.3389/fpls.2022.1046134)

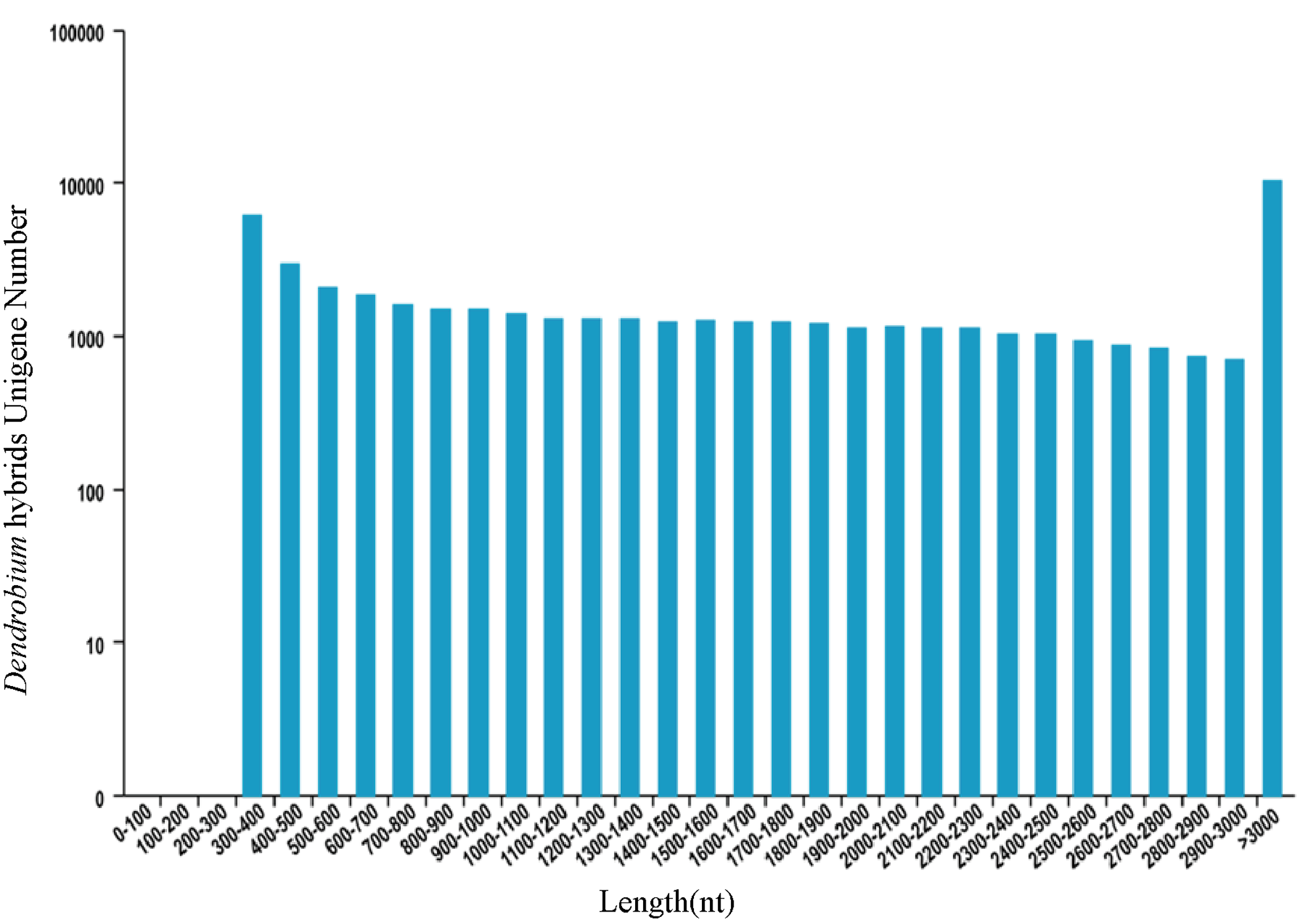

Supplement: Supplementary Figure 1 — Length distribution of unigenes in D. ‘Suriya Gold’. Length distribution of unigenes identified in transcriptome assembly of D. ‘Suriya Gold’. Total number (49,938), Total length (96,387,028 nt), Mean length (1930.13 nt), N50 length (2837 nt). N50 is defined as the length of the longest unigene such that all unigenes of at least N50 length contain greater than 50% of all bases in the transcriptome assembly. [file Image_1.tif]

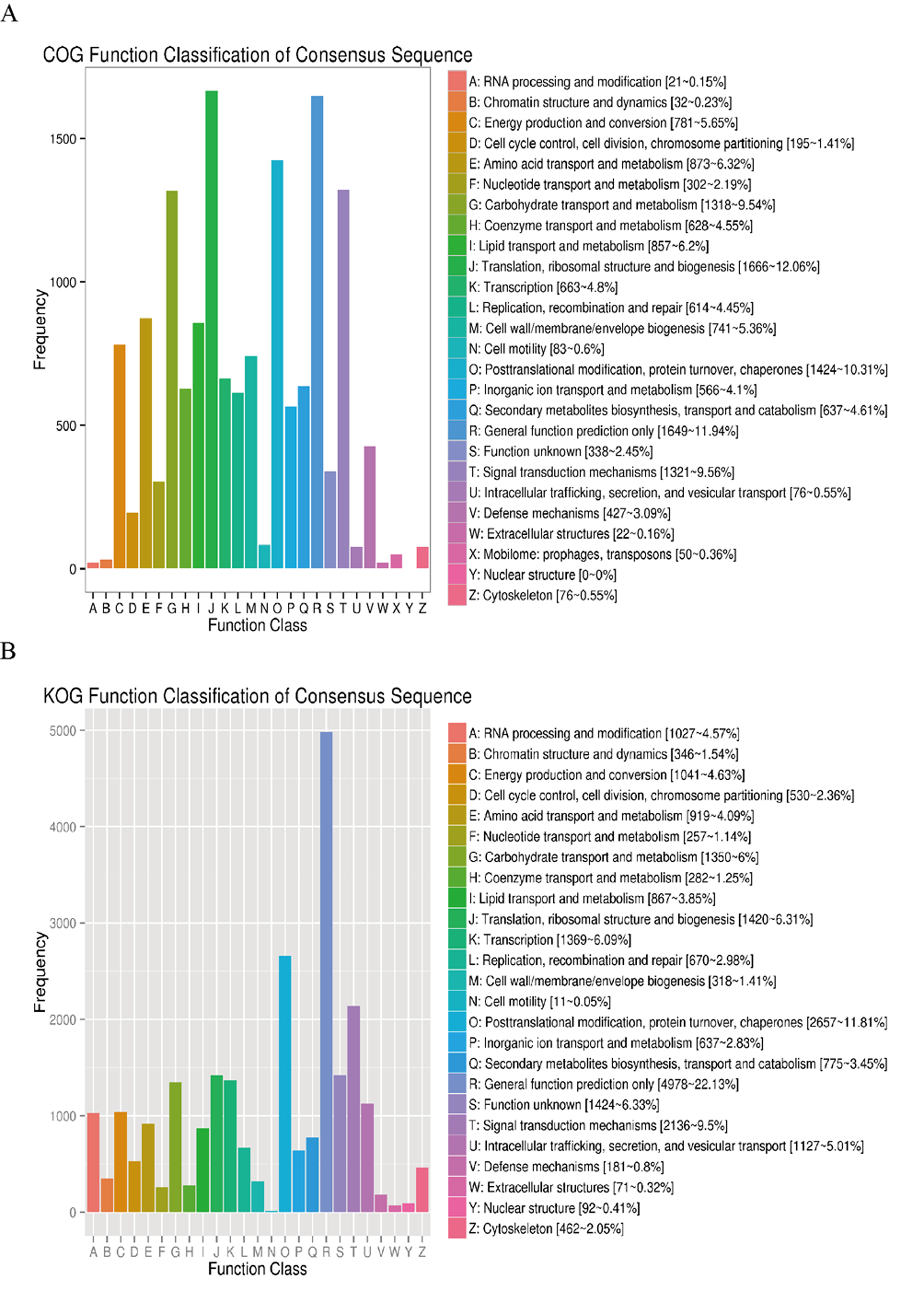

Supplement: Supplementary Figure 2 — COG and KOG functional classifications of unigenes in D. ‘Suriya Gold’. (A) COG (Cluster of orthologous groups) functional classifications of unigenes. (B) KOG (Eukaryotic Ortholog Groups) functional classifications of unigenes. The x-axes represent COG or KOG categories, and the y-axes represent the number of unigenes annotated. [file Image_2.tif]

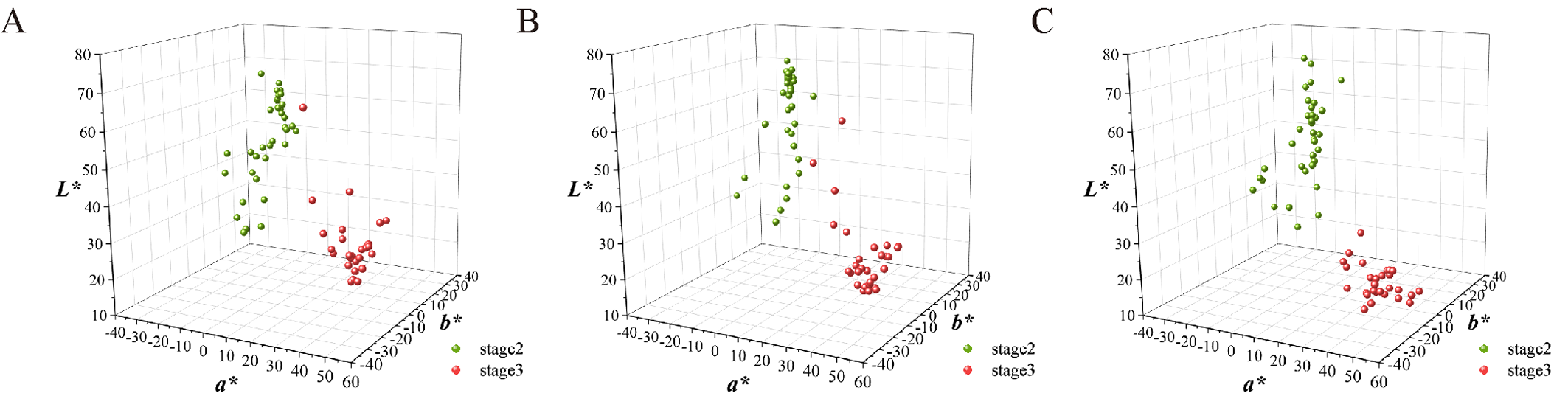

Supplement: Supplementary Figure 3 — Analyses of color in different floral tissues of “BP” hybrid progenies during flower development. Colorimetric analyses of sepals (A), petals (B) and lips (C) of “BP” hybrid progenies at different developmental stages in three-dimensional color space using the CIE L*a*b* system. [file Image_3.tif]

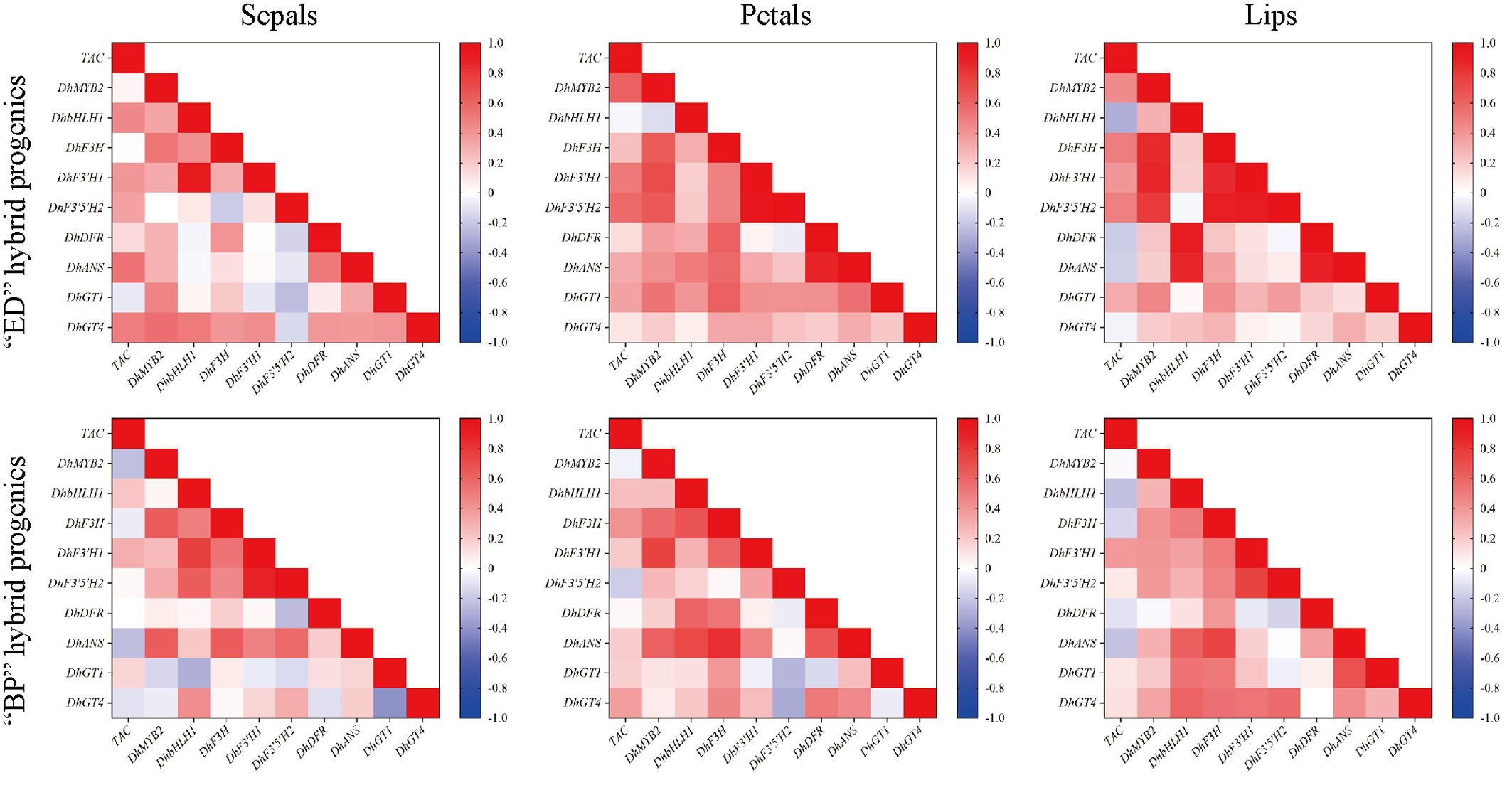

Supplement: Supplementary Figure 4 — Correlation analyses between anthocyanin biosynthesis related genes and anthocyanin content in different floral tissues of two Dendrobium hybrid families. Correlation matrix analyses between anthocyanin biosynthesis related genes and total anthocyanin content (TAC) in different floral tissues (sepals, petals and lips) in hybrid progenies from “ED” or “BP”, respectively. Twenty F1 progenies of each hybrid family were sampled. [file Image_4.tif]
