# Supplementary material for: DhMYB2 and DhbHLH1 regulates anthocyanin accumulation via activation of late biosynthesis genes in Phalaenopsis-type Dendrobium
Source: Front Plant Sci. 2022 Nov 15;13:1046134. doi: 10.3389/fpls.2022.1046134 (PMC9705975; doi:10.3389/fpls.2022.1046134)
Supplement: Supplementary file 5 [file DataSheet_1.docx]

**Table S1 Statistics of Unigene Notes**

| Anno_Database | Annotated_Number | 300bp≼length≼1000bp | length>=1000 |
| --- | --- | --- | --- |
| COG_Annotation | 14007 | 2272 | 11735 |
| GO_Annotation | 18237 | 4715 | 13522 |
| KEGG_Annotation | 15869 | 3474 | 12395 |
| KOG_Annotation | 22495 | 4244 | 18251 |
| Pfam_Annotation | 28566 | 5111 | 23455 |
| Swissprot_Annotation | 27641 | 5587 | 22054 |
| eggNOG_Annotation | 36078 | 7622 | 28456 |
| nr_Annotation | 38653 | 8995 | 29658 |
| All_Annotated | 38833 | 9147 | 29686 |

**Table S2a Correlation analysis of anthocyanin content and *L^*^*, *a^*^*, *b^*^* in “ED**” **hybrid progenies**

| Flowering stages | Flower organs | *L*^*^ value | *a*^*^ value | *b*^*^ value |
| --- | --- | --- | --- | --- |
| Stage2 | Sepals | -0.423 | 0.585^*^ | -0.590^*^ |
|  | Petals | -0.437 | 0.706^**^ | -0.727^**^ |
|  | Lips | -0.579^*^ | 0.544^*^ | -0.578^*^ |
|  | Comprehensive correlation | -0.405^**^ | 0.628^**^ | -0.631^**^ |
| Stage3 | Sepals | -0.819^*^ | 0.669^*^ | -0.475 |
|  | Petals | -0.633^**^ | -0.128 | 0.448 |
|  | Lips | -0.331 | 0.110 | 0.010 |
|  | Comprehensive correlation | -0.475^**^ | 0.311^*^ | -0.120 |

**Note: ^*^, p < 0.05; ^*^ ^*^, p < 0.01.**

**Table S2b Correlation analysis of anthocyanin content and *L^*^*, *a^*^*, *b^*^* in *“*BP**” **hybrid progenies**

| Flowering stages | Flower organs | *L*^*^ value | *a*^*^ value | *b*^*^ value |
| --- | --- | --- | --- | --- |
| Stage2 | Sepals | -0.628^**^ | 0.657^**^ | -0.794^**^ |
|  | Petals | -0.289 | -0.185 | -0.299 |
|  | Lips | -0.419 | -0.260 | -0.08 |
|  | Comprehensive correlation | -0.366^**^ | 0.065 | -0.482^**^ |
| Stage3 | Sepals | -0.570^*^ | 0.394 | -0.532^*^ |
|  | Petals | -0.785^**^ | 0.409 | -0.540^*^ |
|  | Lips | -0.266 | 0.612^**^ | -0.699^**^ |
|  | Comprehensive correlation | -0.600^**^ | 0.391^**^ | -0.467^**^ |

**Note: ^*^, p < 0.05; ^*^ ^*^, p < 0.01.**

**Table S3 The information of key genes and primer sequences used in qRT-PCR**

| Gene name | Unigene No. | Accession No. | Forward primer sequence (5'-3') | Reverse Primer sequence (5'-3') |
| --- | --- | --- | --- | --- |
| *DhF3H* | 095285 | KY399858 | CAAACTCCTCAGCGTGCTCTC | TGAAGCAGGAGGGTGATGGT |
| *DhF3'H1* | 091787 | KY399859 | GAGCACAGTGGAATGGGCTT | AAGGTTGGGGAGATCGGACT |
| *DhF3'5'H2* | 171346 | KY399862 | AGCGTTTTGCACTGTGCTCA | AACGACGCCTTCGACCTTG |
| *DhDFR* | 090298 | KY399863 | TGGAGTGTTCCACGTCGCTAC | ATATCACTCGCTGAACGCTGC |
| *DhANS* | 061266 | KY399864 | GGGAGTGATGCTGCTTGTGAA | TCCTGCCACTCAAGTTTACCG |
| *DhGT1* | 290210 | KY399865 | TTTGGCGACAGGAACTCTAAA | GTGGAGAGAGGCGGTTGAGT |
| *DhGT4* | 073055 | AB692769.1 | TCCAGATAAGAGCAATGGCGA | GGAGAAGCCTTGACCAAGAAAT |
| *DhMYB2* | 185592 | KY039157 | TGATTGCTGGAAGGCTACCC | TCTTTGTATGATGCTTGGGCG |
| *DhbHLH1* | 070225  /290515 | KY039158 | AGCTCATAAATGCAGCAGTGTCC | ATCATGCACCATGGTAGCATCTG |
| *Actin* | - | KC171011.1 | TATTGTGCTTGATTCTGGTGATG | GACAATTTCACGCTCTGCAGTAG |

| Primer names | Sequences of primers (5'-3') |
| --- | --- |
| DhF3'H1-1-SP1 | GCGTGTAGCGTCTGGTGAGG |
| DhF3'H1-1-SP2 | CGGAAGGTTCCCGATGATCG |
| DhF3'H1-2-SP1 | GCGTGTAGCGTCTGGTGAGG |
| DhF3'H1-2-SP2 | GGAATTCAATCTGCAACAGCTG |
| DhF3'H1-3-SP1 | GCGTGTAGCGTCTGGTGAGG |
| DhF3'H1-3-SP2 | GTGGTTTGAGAGTATGAAGAG |
| DhF3'5'H2-1-SP1 | GATGCCCATCTTGAGGAACATG |
| DhF3'5'H2-1-SP2 | CTCCGATGATGGGGAGGTTG |
| DhF3'5'H2-2-SP1 | GATGCCCATCTTGAGGAACATG |
| DhF3'5'H2-2-SP2 | AGTCTCACTATGAGTTTCAC |
| DhDFR-1-SP1 | GGCAGATCCAACAATGGCTTC |
| DhDFR-1-SP2 | GATTTGTTGGATCTCTCACTG |
| DhDFR-2-SP1 | GGCAGATCCAACAATGGCTTC |
| DhDFR-2-SP2 | TTGCCTCATTGGACTTACTG |
| DhANS-SP1 | CGTTATTGAGGGCGTCTACG |
| DhANS-SP2 | TCTACGCGTTCAGAGTCTGC |
| DhGT4-SP1 | TCTGCCACTGCTCCTTCATACTGCA |
| DhGT4-SP2 | AGAGACCTGGTAAGCAGAAGTCCCT |
| DhGT4-SP3 | AGTTGGTACAGCAGGCCTTCTGTGA |

**Table S4 Specific** **primer sequences used in chromosome walking**
